# Review on Sensing Technology Adoption in the Construction Industry

**DOI:** 10.3390/s21248307

**Published:** 2021-12-12

**Authors:** Mona Arabshahi, Di Wang, Junbo Sun, Payam Rahnamayiezekavat, Weichen Tang, Yufei Wang, Xiangyu Wang

**Affiliations:** 1 School of Design and Built Environment, Curtin University, Perth, WA 6102, Australia; Mona.Arabshahi@curtin.edu.au (M.A.); 20760096@student.curtin.edu.au (Y.W.); 2 School of Civil Engineering, Chongqing University, Chongqing 400045, China; 202116131002@cqu.edu.cn (D.W.); 202116131275@cqu.edu.cn (W.T.); 3 Institute for Smart City of Chongqing University in Liyang, Chongqing University, Liyang 213300, China; tunneltc@gmail.com; 4 School of Built Environment, Western Sydney University, Penrith, NSW 2751, Australia; p.zekavat@westernsydney.edu.au

**Keywords:** sensing technologies, construction automation, construction performance, radio frequency identification, ultra-wideband technology, fiber optic sensing technology

## Abstract

Sensing technologies demonstrate promising potential in providing the construction industry with a safe, productive, and high-quality process. The majority of sensing technologies in the construction research area have been focused on construction automation research in prefabrication, on-site operation, and logistics. However, most of these technologies are either not implemented in real construction projects or are at the very early stages in practice. The corresponding applications are far behind, even in extensively researched aspects such as Radio Frequency Identification, ultra-wideband technology, and Fiber Optic Sensing technology. This review systematically investigates the current status of sensing technologies in construction from 187 articles and explores the reasons responsible for their slow adoption from 69 articles. First, this paper identifies common sensing technologies and investigates their implementation extent. Second, contributions and limitations of sensing technologies are elaborated to understand the current status. Third, key factors influencing the adoption of sensing technologies are extracted from construction stakeholders’ experience. Demand towards sensing technologies, benefits and suitability of them, and barriers to their adoption are reviewed. Lastly, the governance framework is determined as the research tendency facilitating sensing technologies adoption. This paper provides a theoretical basis for the governance framework development. It will promote the sensing technologies adoption and improve construction performance including safety, productivity, and quality.

## 1. Introduction

Data collection is crucial in management owing to the complexity and dynamic nature of construction projects [1,2]. However, the conventional data collection process cannot meet the increasing requirements of modern construction management due to the defects in automation and cost [3]. Thereby, Automated Data Collection (ADC) methodologies are in great demand, which could reduce human errors and benefit projects on planning, procurement, control, construction, and management [4,5]. As a base for ADC methodologies, sensing technology is promising in improving data collection and ongoing monitoring [6]. To a large extent, sensing technology can instantly collect and permanently store environment data, revolutionizing data collection, transmission, and analysis in the construction industry. Meanwhile, sensing technology introduces innovative technologies including Information Technology (IT) and digital construction sites, in which sensing technologies promote construction safety and productivity.

Sensing technologies have been academically researched, but their adoption has been challenged by technology-related, adoption process-related, and human-related factors [7,8]. First, inadequate understanding and neglect of automatic sensing technologies restrict real project adoption, despite great benefits in securing construction safety [9]. Taneja et al. [10] claimed the necessity of educating construction practitioners on sensing technologies. Second, information on in-use sensing technologies such as Real-Time Locating Systems (RTLS) is insufficient [11,12]. Specifically, the deployment, time, cost, and accuracy of sensing technologies need to be emphasized [13,14,15]. Third, perceptions of construction stakeholders toward sensing technologies and decision-making criteria for their adoption are scarce [16]. Odubiyi et al. [7] concluded human-related factors were critical in construction adoption, highlighting the perceptions from construction stakeholders. Fourth, research on the acceptance of sensing technologies by construction workers is insufficient. According to the previous research on ADC, acceptance of sensing technologies by construction workers has been ignored in the human-related factors [4]. Fifth, Sepasgozar et al. [17] reported that a thorough understanding of procedures to introduce new technologies into existing systems is a critical factor to facilitate the adoption of sensing technologies in construction. In summary, the current status of sensing technologies and factors restricting their adoption are not yet clear.

This review investigates the current status of sensing technologies and explores the factors influencing their adoption. Previous research mostly focuses on one single group of technologies. Schall et al. [18] have studied the barriers to the adoption of wearable sensors in the workplace. In addition, Usman et al. [19] have analyzed the information and communication technology innovation for construction site management. However, this paper was dedicated to general sensing technologies rather than specific types of sensors. To begin with, common sensing technologies are classified and reported according to their features on safety, quality, and productivity. Of 187 potential articles on types of sensing technologies and their applications in construction, 127 were selected to classify technologies based on their applications. Additionally, their applications and limitations are carefully studied to understand their application status. Then, factors influencing the adoption of various types of digital technologies are extracted from construction stakeholders’ perceptions about the demand, benefits, suitability, and barriers of such technologies. Compared with previous research on ADC, acceptance of sensing technologies by construction workers is added to the review of the human-related factors [4]. Of 69 articles relevant to the adoption of technology, 47 were subsequently analyzed to identify factors affecting the adoption of sensing technologies in construction. Finally, a conclusion is summarized that the governance framework is in great demand for facilitating sensing technology adoption. The governance framework highlights the sensing technology benefits, decision-making considerations, and construction-specific expectations while specifying barriers to deal with. In future research, influential factors uniquely related to sensing technologies and common factors between sensing technologies and other digital technologies need to be separated. This review will facilitate the sensing technologies adoption then improve the construction industry on safety, quality, and productivity. 

## 2. Current Status of Sensing Technologies in Construction

### 2.1. Methods and Material for Literature Review 

**Figure 1:**
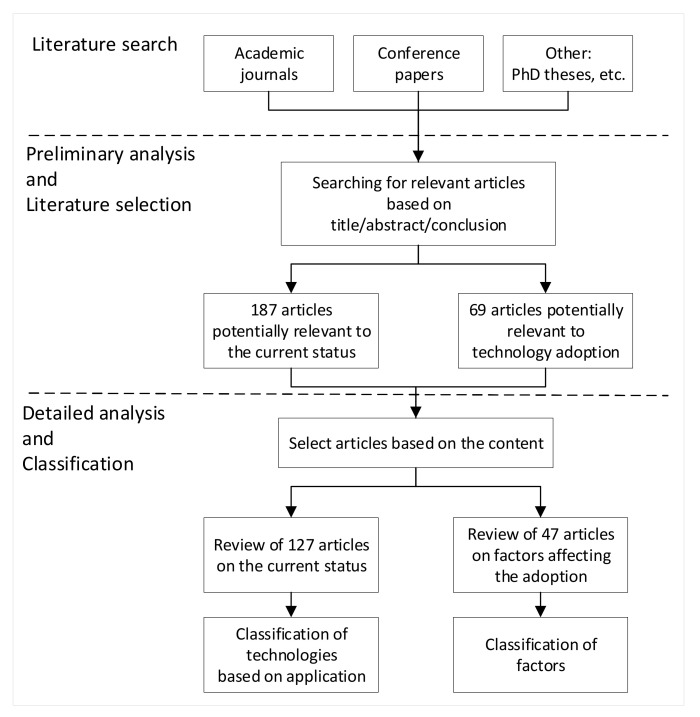
Method for literature review.

The method used for the literature review took place in seven sequential steps. These steps are “scope definition and clarification”, “a literature search” to find potential relevant resources, “a preliminary literature analysis” and “relevant literature selection” to identify and shortlist relevant literature, “detailed literature analysis” to extract related materials, “classification of the findings” for the sake of easy reporting, and finally, “reporting the results”. The process followed for the literature review in this chapter is presented in Figure 1, which also specifies the number of articles identified and shortlisted for both the current status of sensing technologies in construction and the factors affecting their adoption. Of 187 potential articles on types of sensing technologies and their applications in construction, 127 were selected to classify technologies based on their applications. Of 69 articles relevant to the adoption of technology, 47 were subsequently analyzed to identify factors affecting the adoption of sensing technologies in construction.

### 2.2. Sensing Technologies in Construction Safety Enhancement

#### 2.2.1. Location-Based Sensing Technologies

**Figure 2:**
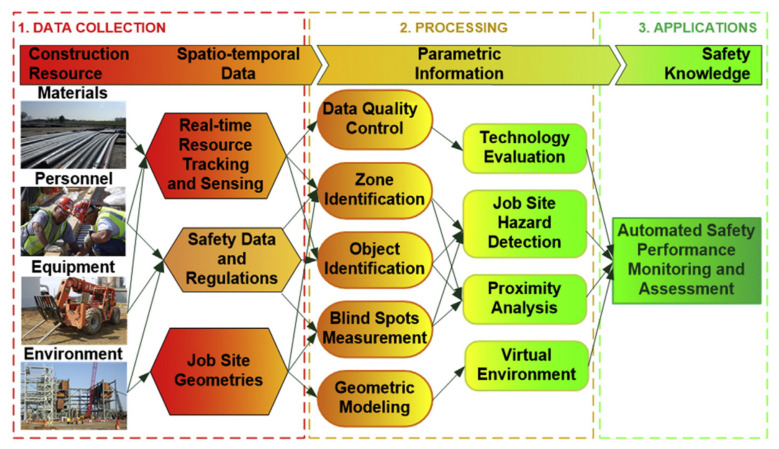
Data collection, information processing, and application framework of RTLS [26].

Location-based sensing technologies, also known as Real-Time Locating Systems (RTLS), are based on wireless technologies including Wi-Fi, Bluetooth, Global Positioning System (GPS), Radio Frequency Identification (RFID), and ultra-wideband (UWB) technology. RTLS are effective in construction management on the process, safety, and on-site resource through locating and tracking construction materials [12,20]. These systems are also effective in situational awareness enhancement [21,22], quantitative hazard exposure analysis [23,24], and behavior-based safety monitoring [25]. The data collection, information processing, and application framework of RTLS are presented in Figure 2. However, they still have shortcomings such as weak signal, high cost, and low accuracy [14]. The applications and limitations of the most common technologies are investigated as follows.

##### Global Positioning System (GPS) Technology

GPS is the most prevalent location-based sensing technology realizing powerful capabilities through satellites [1]. The main components contain the space segment, the control segment, and the user segment. The space segment sets 24 satellites, at least four of which must be visible from a given point on the Earth at any time for most applications. The control segment consists of one master control station, five monitor stations, and four ground antennae to track the satellites and calibrate the clocks. The user segment is made up of the user using a GPS receiver to determine his location based on a received signal. Great improvements resulting from GPS in construction safety are summarized in Table 1. However, the GPS application is also hampered by data delays in transmission, low performance in congested areas, and signal blockage in indoor environments [6,27]. 

##### Radio Frequency Identification (RFID) Technology

**Figure 3:**
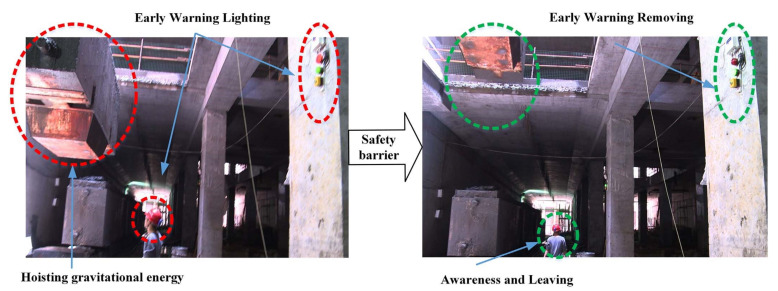
Hazard energy monitoring and safety barrier response in the tunnel shaft area [41].

RFID technology identifies objects through radio waves, reading digital data encoded in RFID tags without direct contact or line-of-sight. The advanced technology is more efficient in tracking materials and equipment compared to traditional barcode systems [37]. An RFID system usually consists of a reader transmitting radio waves, radio frequency tags attached to items, and a software system managing collected information [38]. Furthermore, the RFID system obtains versatile tag categories to satisfy construction demands. An active tag equips a built-in power source enabling the tag to transmit data on its own. Passive tags are more popular than active tags as they are smaller and cheaper. RFID technology presents great performance on both outdoor and indoor construction projects, where satellite position information is unavailable [39,40]. For instance, RFID technology can secure construction safety when the gate crane driver cannot observe the workers at the bottom of the tunnel shaft [41]. The RFID tags on helmets and RFID readers around this area will generate safety warnings for the potential hazard during vertical transportation (Figure 3). Then, the nearby workers would be informed to leave this hoisting area. Other utilities of RFID technology in reducing safety risks are summarized in Table 2. The RFID system has been identified as the most popular sensing technology among all RTLS, although its adoption is still slow [37]. Some limitations restrict the RFID technology application including simultaneous identification of multiple tags and range issues due to metal obstacles.

##### Ultra-Wideband (UWB) Technology

**Figure 4:**
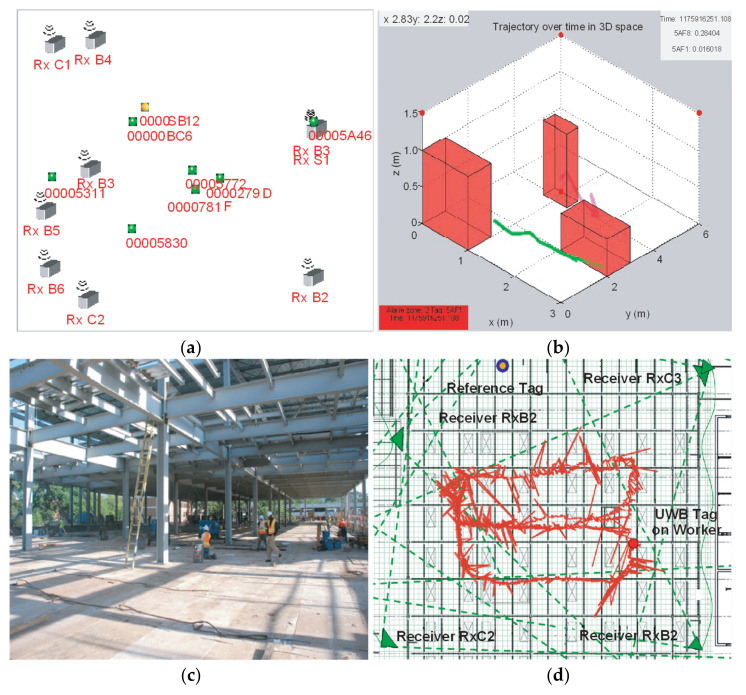
Experiments for obstacle avoidance and field personnel tracking: (**a**) receiver (Rx…) and tag (00…) layout during field trial; (**b**) predefined hazardous areas, UWB path, and alarm box; (**c**) steel structure in field trial; and (**d**) unfiltered UWB data on the trajectory of a worker [54].

UWB technology monitors construction resources and materials by high-bandwidth radio communications. The advanced technology has been used in locating hazard zones, avoiding collision, and increasing situational awareness [51,52,53]. Teizer et al. [54] validated the outstanding functions of UWB technology in obstacle avoidance and field personnel tracking. Experiments employing UWB technology in workforce and materials monitoring is demonstrated in Figure 4. UWB technology exhibits great performance in interior construction sites and harsh environments, even where wooden materials block signals [55,56,57]. However, metal blockage reduces the effect of UWB technology [58,59]. The function of UWB technology is also weakened by range issues over long distances, missing data, possible calibration difficulties, and limited update rates [6].

#### 2.2.2. Vision-Based Sensing Technologies

**Figure 5:**
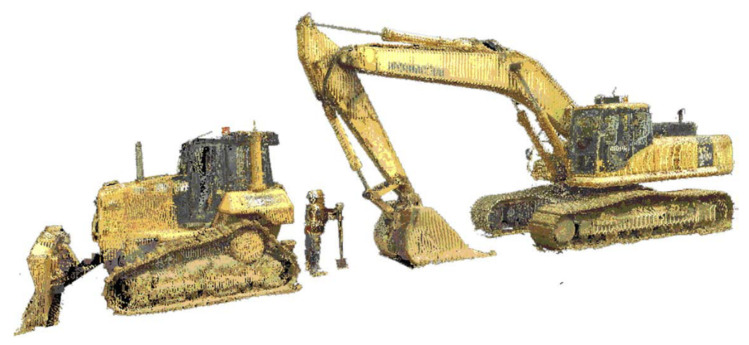
Hazardous situation of worker and equipment measured by a 3D laser scanner [66].

Vision-based sensing technologies range from well-established technology photographs and video recordings to contemporary technology laser scanning, benefit safety management. Photographs and video recordings technology promotes decision making of operations, blind lifts of tower cranes, and communication between project network and work-front operations [60,61,62]. Laser scanners capture detailed geometries and environmental conditions through laser signals emitted from a rotating laser photon source [10]. The scanners are used in intensifying situational awareness of crane operators [21], simulating construction sites [63], and monitoring the construction activities [11,64,65]. The hazardous situation of workers and equipment measured using a 3D laser scanner is demonstrated in Figure 5. However, laser scanning technology is not suitable for moving objects or providing information about colors, textures, and materials. Other factors also restrict the implementation, including a clear line-of-sight requirement, long data processing time, and high data storage capacity.

#### 2.2.3. Wireless Sensor Networks (WSN) Technologies

WSN technologies enable wireless communication between sensors and data recording devices. Common sensors applied in WSN to improve construction safety management include temperature sensors, displacement sensors, light sensors, pressure sensors, and Fiber Optic Sensing (FOS) [67,68,69]. For instance, FOS technology measures temperature, strain, and pressure by the transmission of light through an optical fiber [70,71,72]. It is completely immune to electromagnetic interference and capable of functioning in hostile surroundings [73,74]. Meanwhile, fiber optic sensors are user-friendly devices with an elevated sensitivity which makes them suitable for detecting crack damage in concrete structures [75,76,77]. Fibre Bragg grating (FBG) sensor is one type of FOS which realizes real-time temperature monitoring and displacement measurement, such as tunnel segments [78,79,80]. The utilization of WSN technology supports construction safety greatly, which is summarized in Table 3.

### 2.3. Sensing Technologies in Occupational Health and Safety (OHS) Enhancement

**Figure 6:**
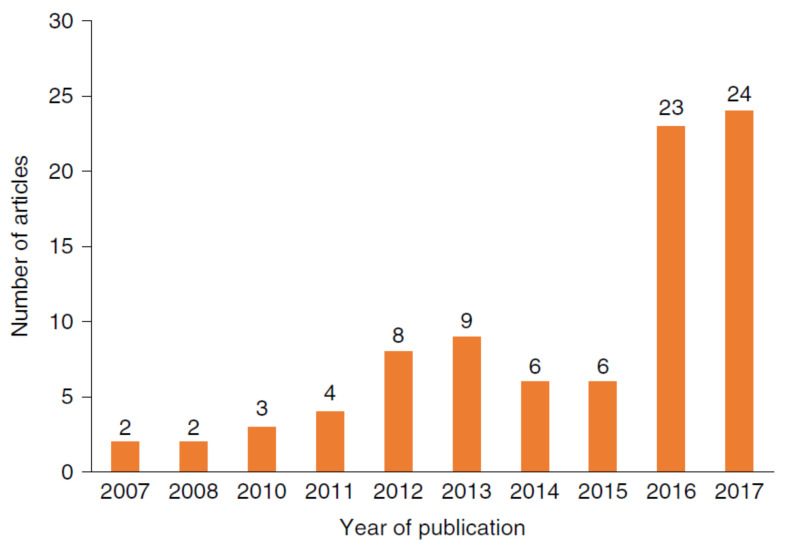
The annual publication trend of sensing and warning-based technology [9].

OHS is a major division of construction safety management in which sensing technologies contribute to remarkable improvements. Articles on sensing and warning technologies have increased exponentially since 2016, indicating that sensing technologies are effective in OHS (Figure 6). OHS is strengthened by vision-based sensing technologies comprising Closed-Circuit Television (CCTV), video cameras, range cameras, and built-in sensors of smartphones [90,91]. Methods of the above technologies to assess the effects of wearing hard hats are demonstrated in Table 4. Additionally, RFID technology secures OHS by checking the presence and compliance of personal protective equipment. Moreover, wearable sensors and environmental sensors have been a focus of research in securing OHS [92]. Wearable devices could simply be available devices such as smartwatches and wristbands which integrate various sensors for monitoring of workers’ health [93,94]. Specific devices such as a chest sensor recording heart rate and heart rate variability are also included in wearable sensing technologies. The effects of wearable sensing technologies in promoting OHS are demonstrated as follows.

#### 2.3.1. Physiological Sensors

Physiological wearable sensing devices benefit OHS through emotional wellbeing monitoring, physical workload and fatigue monitoring, and posture detection [99]. Electroencephalograms (EEGs) monitor stress levels, mental fatigue, emotional states, and attention level [100,101] by tracking and recording brain wave patterns. EEGs provide a base for investigating and addressing any psychological problems of construction workers and, therefore, avoid unsafe behaviors. In addition, electrocardiograms (ECGs) used in chest sensors monitor heart rate and variability of construction workers [102]. Wristband-type heart rate monitoring devices are also used to capture significant variations in physical demands [94,103], estimate energy expenditure [102], and track heart rate [104,105]. Furthermore, ECG, EEGs, and infrared temperature sensors are combined for real-time monitoring of physical fatigue in construction workers [106]. Moreover, surface electromyography monitors the spinal biomechanics of a construction workforce by measuring the electrical activities of muscles. It secures OHS of construction workforces exposed to repetitive lifting tasks and tying rebars [107,108].

#### 2.3.2. Integrated Sensors in Personal Protective Equipment (PPE)

**Figure 7:**
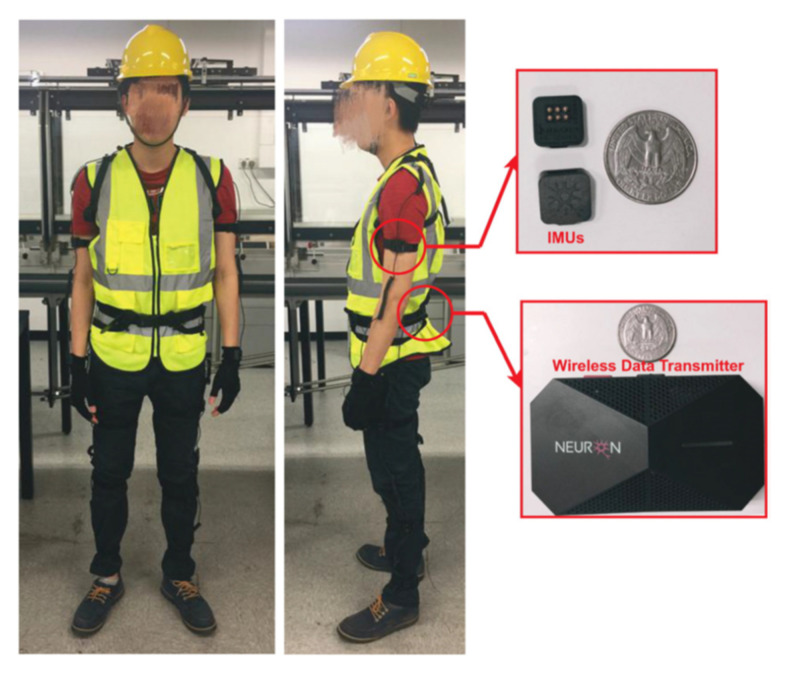
IMU-based wearable motion capture system (Perception Neuron) [111].

**Figure 8:**
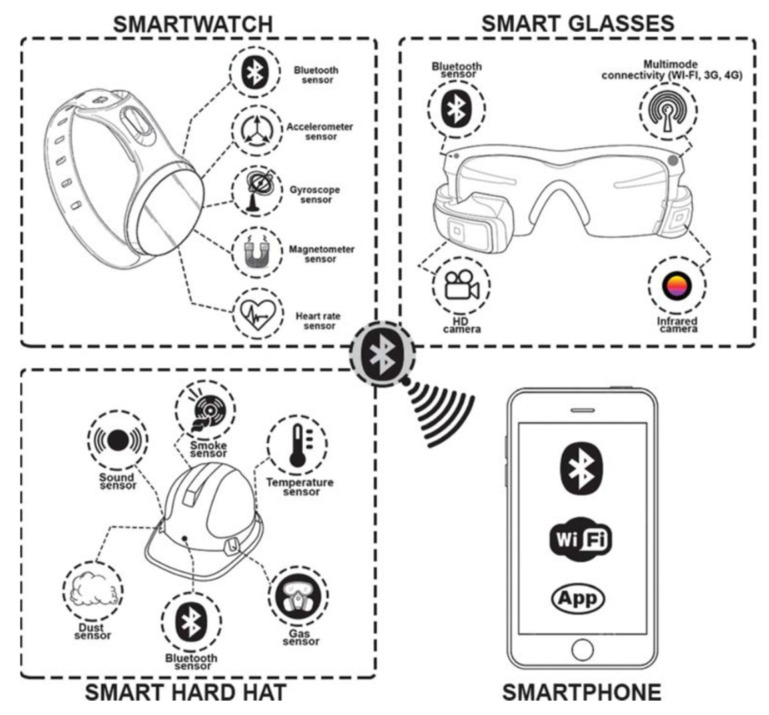
Smart technology and sensors attached in PPE [116].

Wearable sensing technologies attached to PPE realize safety risk detection and health monitoring. To begin with, Inertial Measurement Units (IMUs) are the most common motion sensors in PPE to detect awkward postures [109], gait abnormalities [110], and fall-risk assessments [111]. IMU-based wearable motion capture system (Perception Neuron) used in acquiring experiment data is exhibited in Figure 7. Furthermore, pressure sensors and three-axis accelerometers are valid in assessing the PPE wearing effect [112,113]. Moreover, dust sensors can monitor fine particle concentration and protect workers against excessive respirable dust [114,115]. Lastly, Adjiski et al. [116] proposed a prototype system, which was an outstanding example of different sensors integrated into a system and attached to PPE. In the system, helmets and safety glasses were equipped with sensors connected with smartphones and smartwatches (Figure 8). Sensors used in the system included gas sensors, dust sensors, sound sensors, smoke sensors, temperature sensors, accelerometers, gyroscopes, magnetometers, heart rate sensor measures, and cameras. The prototype system designed to secure OHS during mining operations also meets requirements on other underground construction operations.

### 2.4. Sensing Technologies in Construction Quality Enhancement

**Figure 9:**
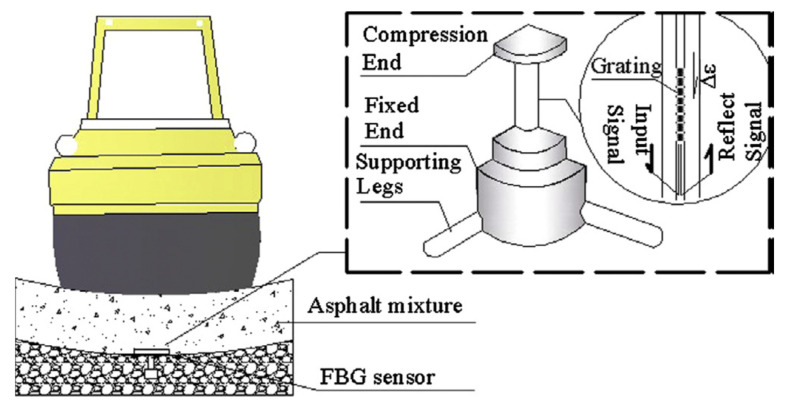
FBG sensor embedded in asphalt pavement [117].

Sensing technology can also improve construction quality management. FBG technology realizes real-time and convenient quality control for asphalt mixture compaction operation during lab experiments (Figure 9). An FBG sensor consists of a compression end, sensing part, fixed end, and supporting legs [117,118]. The compression end is regarded as a load-bearing plate that can make the FBG sensor deform coordinately well with asphalt pavement. The sensing part is made up of a core with gratings and core-protecting materials. Fixed end and supporting legs are used for fixing sensing parts and making the sensor stand stable, respectively. FOS technology is effective in monitoring temperature and stress/strain variation of reinforced concrete structures during construction [119,120,121]. Furthermore, RFID technology facilitates construction quality controls by assisting with monitoring of concrete curing progress and material quality assurance [122,123]. Moreover, temperature sensors measure the internal temperature of in-place concrete in real-time during the early curing stages and assess the strength [124]. Finally, laser scanning technology prevents the failure of precast concrete elements by identifying deviations of prefabricated modules and assessing precast concrete quality [125,126].

### 2.5. Sensing Technologies in Construction Productivity Enhancement

Construction productivity benefits from optimizing scheduling, cutting back on construction time and cost, and reducing construction waste. Real-time progress reporting of construction activities can overcome cost overruns and scheduling delays. However, conventional data collection processes are labor-intensive, costly, and error-prone. Remote sensing technologies have been proposed to achieve an automated data acquisition platform to improve construction productivity.

#### 2.5.1. Location-Based Sensing Technologies to Improve Productivity

**Figure 10:**
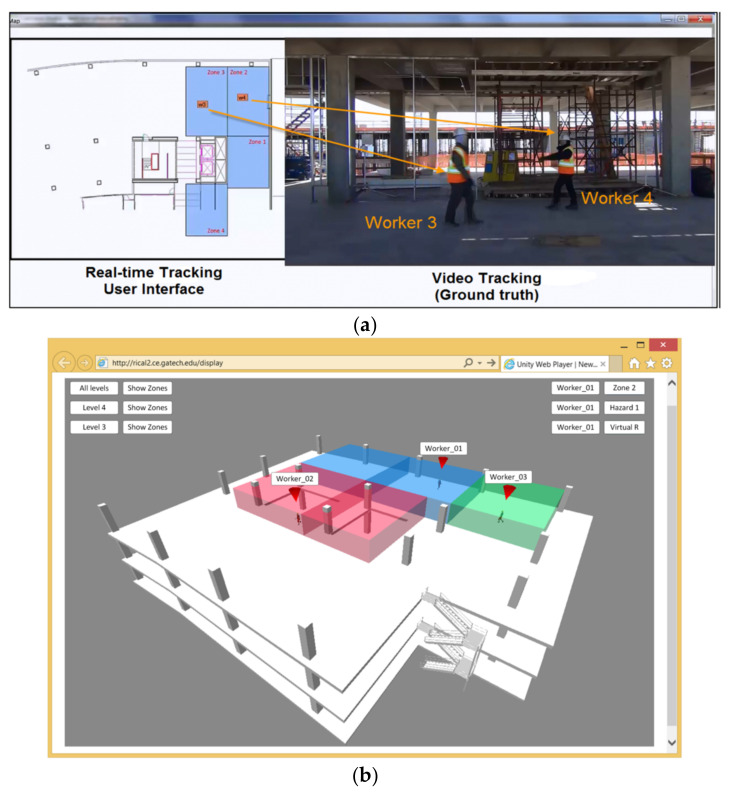
(**a**) recognition rate evaluation by comparing localization results to video recording; (**b**) cloud-enabled remote monitoring user interface [48].

GPS technology obtains multiple potential applications comprising resource localization and materials tracking to increase construction productivity [127,128,129]. For instance, GPS technology collects and provides real-time information of a delivery fleet to reduce productivity loss and idleness [59,130]. Similarly, position tracking of key personnel is also achieved through RFID technology to save cost and time [131,132]. Fang et al. [48] concluded that the BIM and cloud-enabled RFID indoor localization solution had great potential in asset management and productivity monitoring. The cloud-enabled remote monitoring user interface is demonstrated in Figure 10. Moreover, GPS and RFID technologies are combined to realize automated tracking of construction resources, which is beneficial in construction productivity monitoring [133]. Applications of RFID technology in increasing construction productivity are demonstrated in Table 5.

#### 2.5.2. Vision-Based Sensing Technologies to Improve Productivity

**Figure 11:**
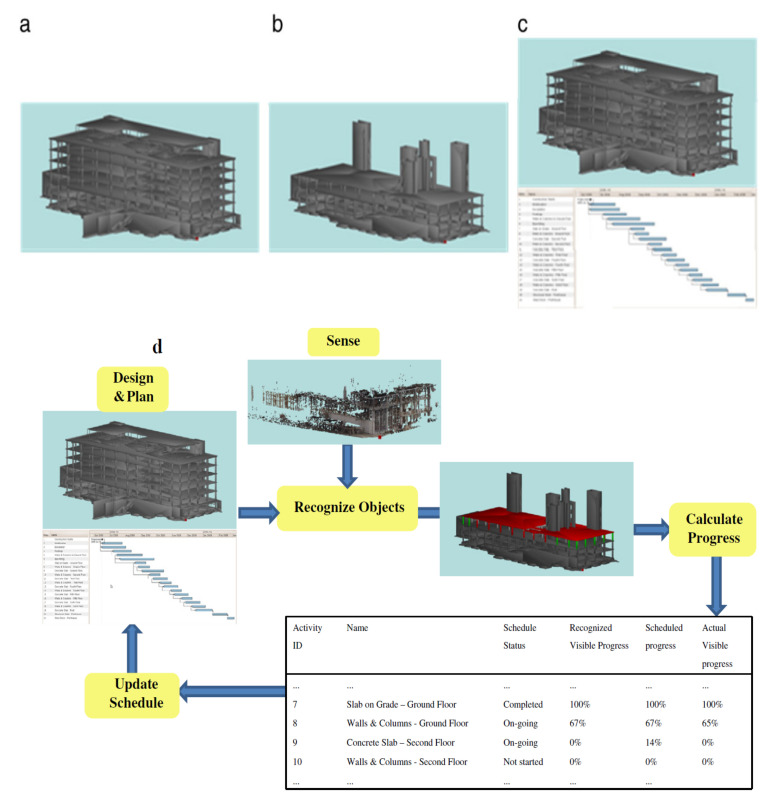
(**a**) 3D model, (**b**) time-stamped 3D model, (**c**) 4D model, and (**d**) procedure for automated progress calculation and schedule update [149].

Video cameras can monitor and track construction resources to improve construction productivity [147,148]. Three-dimensional (3D) laser scanning technology combined with schedule information can result in more effective and efficient tracking of construction progress than manual works [149]. 3D point clouds, project 3D computer-aided design (CAD) model, and schedule information are combined to track construction progress. First, 3D laser scanning data provides current site conditions. Second, the 3D CAD model combined with schedule information (the project 4D model) provides designed spatial characteristics of the facility under construction over time (Figure 11). A time-stamped 3D CAD model can thus be formed automatically for a given date with such a 4D model. The 3D point clouds and the 4D model must be registered in the same coordinate system to extract useful data for progress tracking. Once registered, as-built objects can be recognized, progress estimated, and the schedule updated all automatically. Moreover, vision-based sensing technologies are also used along with other technologies for a more robust accuracy in material tracking especially in congested and indoor construction sites. Construction productivity is significantly promoted by integrations such as Photogrammetry with GPS [150] or robotic total stations [151], and the incorporation of video recording with UWB [152,153].

## 3. Factors in the Determination of Sensing Technologies Adoption

Sensing technologies lack adoption in real construction projects, though they have been academically explored and proven to yield positive potential. Sepasgozar et al. [154] discovered the adoption process influenced emerging technologies promotion. The process was classified as new technologies identification, existing options cognition, and options comparison. Opinions from construction stakeholders are crucial factors in the process. Therefore, perceptions of construction managers and the acceptance of construction workers toward sensing technologies are in great demand. Emerging technologies will benefit from the investigation of factors determining the adoption process.

### 3.1. Perceptions of Construction Managers toward Sensing Technologies

The decision-making process for adopting new technology is affected by the managers [155]. General awareness about the benefits, capabilities, and effects will contribute to an improved adoption process [156]. Resistance attributes to insufficient understanding and exposure [157]. In contrast, an understanding of market conditions and technology capabilities increases confidence about the adoption. However, sensing technologies have not yet been fully adopted into the construction industry. Perceptions toward other technologies are also worth exploring, such as Information Technology (IT), Information and Communication Technology (ICT), ADC, and Building Information Modeling (BIM) [158,159]. The scope of the investigation is expanded beyond sensing technologies to extract as much information as possible.

#### 3.1.1. Benefits of Sensing Technologies Adoption

Motivations for the construction industry to adopt digital technologies help discover the factors that can promote sensing technologies in construction. One significant motivation for new IT-based technologies is the competitive advantage in the market [160]. Most new technologies are problem-driven and solution-driven. Digital Twins (DT) create a digital replica of a physical object and synchronize data to achieve monitoring, simulating, and optimizing the physical object [161]. The visualization technology presents dynamic and complex information generated by DT, helping construction managers and on-site workers to make better decisions. New technologies also might be forced by external requirements, such as the request of clients, or compliance with regulations [162,163].

Sensing technologies employment can lead to savings both in terms of social costs and health costs. Therefore, future research should cover the relevant contents to contribute to the states and insurance companies to support the technology application. Analysis of the benefits in Table 6 will facilitate the sensing technologies adoption.

#### 3.1.2. Barriers to Sensing Technologies Adoption

Barriers have been identified through the perceptions of stakeholders such as operating cost, lack of well-trained staff, and technology immaturity, etc. Odubiyi et al. [7] divided barriers in ICT into three broad categories related to technology, process, and people. Sardroud et al. [4] classified the barriers to ADC technologies into cost-related, process-related, and technology-related issues. As most barriers are people-related, solutions will be found through an investigation on the factors that construction stakeholders perceive as barriers.

As a major barrier, the capital cost of implementation has been noted repeatedly [166]. Training costs, maintenance costs, and operating costs [164] are also key components in the barriers. Besides cost-related barriers, challenges related to people are also involved, such as a lack of interest and well-trained staff [157,182]. In addition, technical complications such as lack of integrity, durability, and reliability negatively affect innovative technologies [18,183]. Meanwhile, changes in the management process and complications in the construction site also influence technology adoption [182,183]. Analysis of the barriers in Table 7 will promote the adoption of emerging technologies.

### 3.2. Acceptance of Construction Workers toward Sensing Technologies

User acceptance and trust-building are the two key components of the Internet of Things (IoT)-based technologies adoption in OHS [171]. Engineers, operating crews, and fitters are also consulted before adopting new technology, though managerial support affects employees’ intentions to accept a new system [17]. The acceptance of construction workers has been investigated on IT, mobile computing devices, BIM, and wearable sensing technologies [190,191]. Previous research studies, especially on wearable technologies, reported that privacy, security, and confidentiality were major concerns held by construction workers [18,192]. Workers show great willingness to use wearable sensors if data is only collected during working hours [191]. Meanwhile, the top motivation of construction workers to accept wearable sensing technologies is to identify health risks and promote occupational safety [171]. Therefore, perceived usefulness and perceived ease of use are the two concepts dominating the literature on the acceptance of construction workers toward innovative technologies.

Perceived usefulness is the extent users believe that the system will assist them to achieve better performance [193]. It is determined by various factors such as social influence, job relevance, top management support, and benefits at the organizational level [194]. Perceived usefulness has been recognized as a motivation towards using emerging technologies such as BIM [156], scanner technology [11], and wearable sensing technologies [192]. Perceived usefulness has been a determining factor for technology adoption in the context of construction research [195]. Perceived ease of use is another important factor, which is the degree to which the user believes that they can use a system effortlessly and free from difficulties [193]. Perceived ease of use is usually measured through training and technological complexity [194,195,196]. User satisfaction is more influenced by perceived usefulness than perceived ease of use [166]. As the application of wearable sensors is quite new, it is important to research the acceptance of construction workers. Choi et al. [192] investigated factors influencing the adoption of wearable sensing technologies by construction workers. They discovered that “perceived usefulness”, “social influence”, “perceived privacy risk”, and “perceived ease of use” were the major factors determining workers’ acceptance.

## 4. Future Research

The final goal is to develop a governance framework, which could be referred to for easier decision making on the suitability of particular sensing technology. Sensing technologies demonstrate promising potential in construction safety, productivity, and quality enhancement. However, the construction industry lacks an understanding of these advanced technologies. Information about sensing technologies in real projects is scarce. Additionally, the adoption process demonstrates the crucial factors as opinions of stakeholders and workers. This paper embraces a review of factors that affect the adoption and implementation of digital technologies. The future research will identify, extract, supplement, and rank the factors conducted from this review to determine the significant factors in sensing technology adoption. Awareness of the advantages resulting from the sensing technology implementation is crucial to counter the resistance from the construction industry and promote innovative sensing technologies. Therefore, the construction industry needs a governance framework, which contains a core structure depicting the adoption process, from a proposal to its evaluation and approval. Barriers and benefits work in opposite directions during the proposal, while the risk of introducing new devices into the existing systems is also considered. The proposal progresses to detailed evaluation and approval when the totality of benefits, barriers, and all relevant considerations conclude new sensing technology is suitable. During the evaluation, considerations for the suitability of the proposed sensing technology, the whole of life costs, and factors related to people should be justified. The framework will assist with the implementation of sensing technologies during construction processes. In the case study of the governance framework, overall cost-benefit analyses of these technologies in the construction site need to be considered to incentivize their adoption by stakeholders.

## 5. Conclusions

Applications of sensing technologies and the rationale behind their slow adoption have been explored in this paper. This literature review, as opposed to previous reviews, is not limited to a specific group of technologies. The focus is on sensing technologies effectively improving construction performance such as safety, productivity, and quality. Eight popular sensing technologies are selected, including GPS, RFID, UWB, FOS, pressure sensing technology, temperature sensing technology, visual sensing technology, and 3D scanning technology. The benefits, shortcomings, and their application in construction have been reviewed. Even the most popular technologies such as GPS and visual sensing technology were not fully adopted. Despite sensing technologies having been academically explored and proven to yield positive potential, they lack adoption in real construction projects. This review is not only limited to the factors affecting the adoption, but also covered factors affecting the acceptance of sensing technologies by construction workers. Meanwhile, this review embraced all relevant factors reported in the literature regarding the adoption of almost all types of innovative digital technologies such as IT and BIM. In future research, influential factors uniquely related to sensing technologies and common factors between sensing technologies and other digital technologies need to be separated. Furthermore, research should cover the points of stakeholders inside and outside of the construction industry. It is concluded that the capital cost of sensing technology implementation is the strongest barrier to its adoption. Technical barriers, safety concerns, and ethical concerns are also involved in the decision-making process. Aside from financial constraints and challenges related to skill acquisition, another barrier is related to decision makers or end-users who are resistant to change and lack awareness of the benefits of the proposed new technology. Such barriers could be diminished by raising awareness of the benefits and effectiveness of the intended sensing technology. These factors form the basis of the governance framework, which could be referred to for easier decision making on the suitability of particular sensing technology. Safety, productivity, and quality performance of construction processes will benefit from sensing technologies adoption, and the governance framework will promote a more straightforward adoption process.

## Figures and Tables

**Table 1:** GPS technology in construction safety enhancement.

Benefit	Reference
proximity detection of workers on foot and construction equipment	[26]
unsafe proximity detection identification	[28,29]
construction equipment management	[30,31]
situational awareness improvement of on-site workers	[32]
construction resources identification	[33]
enhancement of tower crane navigation systems	[34]
construction equipment activity recognition	[35,36]

**Table 2:** RFID technology in construction safety enhancement.

Benefit	Reference
risky behavior of workers recognition	[41]
accidents and collision prevention	[42,43,44]
proximity detection alert systems	[45]
storage of safety information	[46]
controls of workers and vehicles to specific positions	[47]
indoor localization of mobile and stationary construction resources	[48,49]
detection of construction workers localization	[50]

**Table 3:** Wireless sensor networks in construction safety enhancement.

Benefit	Reference
improvement on a communication platform for tower crane operations	[61,81,82]
environmental and structural health monitoring	[78,83,84]
recognition and detection of construction operation	[85,86]
access control of restricted areas and examination of proper personal protective equipment	[87]
automated monitoring of construction processes	[88,89]

**Table 4:** Methods of vision-based sensing technologies in hard hats detection.

Method	Reference
object detection methods	[95]
movement prediction of workers	[90]
posture estimation and classification	[96]
identification of potential bodily work-related ergonomic risks	[91]
identification of unsafe behavior	[97,98]

**Table 5:** RFID technology in construction productivity enhancement.

Benefit	Reference
construction waste management and machinery maintenance records	[13]
identification of construction material and resources	[33]
recognition of construction staff location	[131]
automatic progress reports	[134,135]
operational cost reduction in precast construction supply chain	[136]
material localization, monitoring, and tracking	[137,138,139]
active information flow between construction progress and material monitoring staff	[140,141]
applications in time and schedule management	[142]
supply network visibility	[143,144]
asset management and supply chain management	[145,146]

**Table 6:** Benefits of digital technologies according to construction stakeholders.

Benefit	Reference
cost reduction	[164,165,166]
time-saving and improved productivity	[167,168,169]
reduced risk of injury and illness	[170]
increase employees’ wellness and satisfaction	[171]
better document quality	[172,173]
better facilities management	[174]
process and performance improvement	[175,176]
improved leadership and decision support systems	[177]
mechanical enhancement on concrete printing	[178,179,180]
improved quality of construction project delivery	[181]

**Table 7:** Barriers to digital technology adoption according to construction stakeholders.

Barriers	Reference	Barriers	Reference
cost-related		people-related	
operating cost	[164]	lack of well-trained staff	[167]
cost of training and employing professionals	[177]	compliance of employees	[166]
cost of maintenance	[184]	legal or ethical concerns	[171]
implementation cost	[185]	resistance to change	[182]
uncertain cost-benefit relation	[186]	company culture	[187]
		lack of government support	[188]
technology-related		other barriers	
operational difficulties	[19,189]	manufacturing requirements	[18]
power supply issues	[174]	change in the process	[182]
data management issues	[177]	site-related issues	[183]
lack of proper IT infrastructure	[182]	temporary nature of construction	[187]
technology immaturity	[183]		

## Data Availability

The data presented in this study are openly available.
